# Perceived Impact of Quarantine on Loneliness, Death Obsession, and Preoccupation With God: Predictors of Increased Fear of COVID-19

**DOI:** 10.3389/fpsyg.2021.643977

**Published:** 2021-03-10

**Authors:** Violeta Enea, Nikolett Eisenbeck, Teodora Carina Petrescu, David F. Carreno

**Affiliations:** ^1^Department of Psychology, Alexandru Ioan Cuza University, Iaşi, Romania; ^2^Department of Personality, Evaluation and Psychological Treatment, Faculty of Psychology, University of Seville, Seville, Spain; ^3^Department of Psychology, Universidad de Almería, Almería, Spain

**Keywords:** quarantine, loneliness, death obsession, COVID-19, God beliefs, existential positive psychology

## Abstract

Most countries are facing the societal challenging need for a new quarantine period due to the increasing number of COVID-19 infections, indicating a second or even third wave of disease. The COVID-19 pandemic has brought to the surface existential issues that are typically less present in people's focal attention. The first aim of this study was to identify some of these existential struggles such as increased feelings of loneliness, death obsession, and preoccupation with God. Secondly, we explored the association of these factors with the increased fear of coronavirus during the quarantine. Data was collected from 1,340 Romanian adults using a cross-sectional web-based survey design in the midst of the national lockdown period of COVID-19. Participants completed measures of COVID-19 related loneliness, death obsession, and preoccupation with God twice; first, thinking about the period before the pandemic, and second, for the current situation during the quarantine. Then, they completed a fear of COVID-19 measure. Participants perceived an increase in the feelings of loneliness, death obsession, and preoccupation with God during the confinement. Furthermore, gender, knowing someone diagnosed with COVID-19, loneliness, death obsession, and preoccupation with God predicted fear of COVID-19. Interestingly, days in isolation did not predict fear of COVID-19 nor were associated with feelings of loneliness. In line with existential positive psychology, these results highlight the importance of policies and interventions targeting the experience of loneliness, spiritual beliefs, and particularly those aimed to promote death acceptance, in order to alleviate intense fear of COVID-19.

## Introduction

Daily increasing deaths and cases of COVID-19 infection have led to worldwide preventive restrictions like quarantine and lockdown as a solution to limit disease spread (Shen et al., 2020). Most countries adopted lockdown as a containment strategy and leaving home was only allowed if strictly necessary. Individuals in a state of confinement were deprived of usual practices, such as visiting family and friends or participating in social gatherings (Reynolds et al., 2008). The quarantine differs from isolation, which applies to individuals diagnosed with the disease (Manuell and Cukor, 2011), and was described as an unpleasant experience for the population (Brooks et al., 2020). It evokes fear of contagion and infecting other people (Reynolds et al., 2008) and concerns about disease and death (Asmundson and Taylor, 2020). The perception of isolation from the rest of society during the prolonged confinement, unexpected interruption of work, and financial losses raised the levels of stress and anxiety (Brooks et al., 2020). However, although these existential struggles are supposed to have emerged because of the COVID-19 pandemic, there is a need for studies reporting how people experience the raise of existential issues such as loneliness and mortality in comparison with the situation before the coronavirus spread. According to existential positive psychology (PP2.0, Wong, 2011), although these existential struggles are linked to increased distress, they can also encourage the adoption of preventive measures and serve as promoters of personal growth (Wong, 2020a,b). This new paradigm in psychology calls for the inclusion of undesirable emotions and events as important factors in the foundation of well-being. In the context of the present pandemic, identifying the specific struggles people are going through, and understanding the deeper fears behind the fear of COVID-19, is a necessary step in this transformative process.

A recent brief review highlighted the role of anxiety as the dominant emotional response to an outbreak (Lima et al., 2020). Although research showed that quarantine can also contribute to stress and anger (Li et al., 2020), fear has been one of the most frequent psychological reactions in individuals during this coronavirus pandemic (Wang et al., 2020). Functional levels of stress and fear as adaptive reactions are natural and useful to the pandemic situation, promoting the protective behaviors (maintaining physical distance, washing hands, etc.) that facilitate COVID-19 prevention (Harper et al., 2020). However, insufficient or severe levels of fear may be maladaptive and detrimental to physical and mental health. Insufficient fear may determine people ignoring the risks of infection and government measures to slow the spread of coronavirus (Harper et al., 2020). On the other hand, high levels of fear are associated with anxiety and depression (Ahorsu et al., 2020), discrimination and xenophobia (Devakumar et al., 2020), and suicide risk (Mamun and Griffiths, 2020). Some studies found that a higher level of COVID-19 related fear may be associated with more intense psychological distress during quarantine (Fernández et al., 2020) and may exacerbate pre-existing mental health disorders (Colizzi et al., 2020). Thus, Brown et al. (2020) have reported cases of heightened anxiety about COVID-19 leading to psychosis and related psychopathology. Furthermore, fear of acquiring COVID-19 disease led to a significant decrease in diagnostic examination (Filice et al., 2020) that have caused a delay in the diagnosis of acute cardiovascular disease (Baldi et al., 2020) and delayed follow-up of the oncological diseases (Kumar and Dey, 2020).

To our best knowledge, only one previous study investigated possible predictors of increased fear of the coronavirus. Mertens et al. (2020) found that variables like personal relevance (risk for loved ones, risk control, and personal health), psychological vulnerability factors (worry, health anxiety, and intolerance of uncertainty), and media exposure were significant predictors of increased fear of COVID-19. As it is important to establish relevant predictors of increased fear of coronavirus (Broche-Pérez et al., 2020; Pakpour and Griffiths, 2020), in the current study, we examined the contribution of other risk factors with an existential character during quarantine: loneliness, death obsession, and preoccupation with God. A better understanding of the respective influence of these factors on the fear of coronavirus would help identify groups at risk and thus improve interventions to stimulate the individuals' compliance with the protective measures to slow the spread of coronavirus. Moreover, it would help to improve psychological health since coronaphobia is a predictor of psychological distress during the COVID-19 crisis (Lee et al., 2020).

In this work, loneliness is understood as the subjective distress resulting from a discrepancy between the perceived and desired frequency of social relationships (Perlman and Peplau, 1981). The feeling of loneliness has been found to be related to fear and emotional hypervigilance in daily life (Meng et al., 2020), mental health conditions (Ong et al., 2016), increased somatic complaints (Wei et al., 2015), higher levels of anxiety sensitivity (Narchal and McDavitt, 2017), increased depressive symptoms (Cacioppo et al., 2010), and suicide (Stickley and Koyanagi, 2016). A recent study found that COVID-19 related loneliness was also associated with more sleep problems (Grossman et al., 2021). This is in line with the idea that loneliness is one of the main factors that produces existential angst (Yalom, 1980). The current study is the first one that investigated the association between loneliness and fear of COVID-19 during the quarantine.

The mass media, in the form of television and radio, inform people on the exact number of daily coronavirus deaths, on the lethality of the virus, and, thus, individuals may be primed with thoughts of mortality. Terror Management Theory (TMT; Greenberg et al., 1986), inspired by the work of existential theorists (Becker, 1973), is the most influential theoretical approach about how individuals psychologically deal with death. It proposes that reminders of our mortality lead to strong anxiety that individuals are motivated to reduce through defensive mechanisms (Rosenblatt et al., 1989). However, in line with the Meaning Management Theory (MMT, Wong, 2008), which is linked to existential positive psychology, death remainders can also activate life appreciation and a proactive search for spiritual growth. According to this theory, confrontation with death reminders may activate a search for the ultimate meaning of life, typically translated as spirituality (Muldoon and King, 1995). In this context, religious beliefs may provide a buffer against the fear of death and promote death acceptance (Jong et al., 2012). Religiosity facilitates coping with negative events and can also favorably affect mental health through lower stress and less anxiety (Koenig, 2012). For that reason, in the current study, we decided to explore this spiritual search during quarantine by using a subscale of the Dimensions of Religiosity Scale (Diduca and Joseph, 1997) that measures preoccupation with the belief in God.

Different negative emotions and cognitions may be revealed by the expectancy of death. While the research literature on death-related topics is dominated by studies investigating death anxiety (Cohen et al., 2005; Postolică et al., 2019), in the current study, we focused on another component of death distress, namely death obsession (Abdel-Khalek, 2004). This component consists of rumination, repetitive or intrusive thoughts, or images that are centered around the death of self or significant others (Rajabi, 2009; Mohammadzadeh et al., 2018). Although certain levels of preoccupation with death can be adaptive, death obsession may be more indicative of intense death anxiety and death avoidance, that is, the unwillingness to contact with the idea of death (because of panic) which is reflected in excessive mental efforts to maintain a feeling of control about its occurrence. This attitude toward personal mortality can be problematic. For instance, studies showed that death obsession was positively associated with death-related depression, death anxiety, anxiety, depression, and neuroticism (Maltby and Day, 2000). Thus, death obsession may reflect a lack of death acceptance (understood as being at ease with the awareness of death) that is linked to lower levels of death fear and anxiety (see Wong et al., 1994). So far, little research has investigated the relationship between death obsession and coronavirus perceived risk (Yildirim and Güler, 2021), while no study examined the association between death obsession and pandemic-specific stressors such as fear of COVID-19 during the quarantine.

The primary aim of this study was to identify potential risk factors associated with fear of coronavirus that are especially relevant during this pandemic from an existential psychology point of view, such as loneliness, death obsession, and preoccupation with God. To better understand the effect of the pandemic context, we measured these factors twice. First, participants reported their condition before the pandemic retrospectively. Second, they responded based on their current status, during the quarantine. Given the controversy regarding the factor structure of the Fear of COVID-19 Scale (FCV-19S) (Pakpour et al., 2020) developed by Ahorsu et al. (2020), we additionally assessed the psychometric characteristics of the Romanian version of the FCV-19S (confirmatory factor analysis and invariance testing) to enhance its utility for use in research and practice. We hypothesized that: (1) levels of loneliness, death obsession, and preoccupation with God would be higher during quarantine than before the COVID-19 pandemic; (2) loneliness, death obsession, and preoccupation with God during quarantine would be positive predictors of fear of COVID-19, (3) based on previous studies (Bitan et al., 2020; Savitsky et al., 2020), gender would be a moderator of the relationship between loneliness and fear of COVID-19, and (4) loneliness would be a moderator of the relationship between death obsession and fear of COVID-19.

We finally discuss how existential positive psychology (Wong, 2011, 2020a,b) offers an integrative approach to treat these existential struggles and use them as promoters of personal growth and resilience.

## Methods

### Participants

Initially, 1,392 people agreed to participate in the study. Underage participants (six cases), individuals diagnosed with COVID-19 (six cases), and outliers with 3 SD (41 cases) were removed. The final sample thus consisted of 1,340 Romanian adult participants, with 1,125 (84%) females. Age ranged between 18 and 71, with an average of 38.83 years old (*SD* = 10.86).

### Measures

All demographic data was dichotomously categorized (e.g., male/female, student/working, maintained activities/not, illness/not illness, know someone infected with coronavirus/not know someone.

### Fear of Coronavirus Scale

The Fear of Coronavirus Scale (FCV-19S; Ahorsu et al., 2020) is a seven-item scale that measures the severity of COVID-19 perceived fear (e.g., “*My heart races or palpitates when I think about getting coronavirus-19*”). Participants indicate their level of agreement on a 5-point Likert scale from (1)”*strongly disagree*” to (5)” *strongly agree*.” The higher a participants' total score is, the greater is the fear of COVID-19. Cronbach's alpha was 0.92 in the present study.

### UCLA Loneliness Scale Version 3

UCLA Loneliness Scale Version 3 (Russell, 1996) is a 20-item scale that measures an individual's feeling of loneliness and feeling of social isolation (e.g., “*How often do you feel that you lack companionship?*”). Subjects answered each item on a scale from 1 (*never*) to 4 (*often*). Higher scores represent a more pronounced perception of loneliness and social isolation. Cronbach's alpha for the scale was 0.92 for the present situation and 0.94 for the past situation in the present study.

### Death Obsession Scale

The Death Obsession Scale (DOS; Abdel-Khalek, 1998) is a 15-item self-report measure of an individual's obsessive thoughts in connection with death (e.g., “*The idea that I will die dominates me*”). Participants indicate their answer on a five-point Likert scale, answers ranging from “*no*” (1) to “*very much*” (5). Higher scores indicate more present obsessive thoughts about death. Cronbach's alpha for the scale was 0.96 for the present situation and 0.96 for the past situation in the present study.

### The Dimensions of Religiosity Scale

The Dimensions of Religiosity Scale (Joseph and Diduca, 2007) is a 20-item scale that contains items divided into four dimensions: preoccupation, guidance, conviction, and emotional involvement. For the current study, we only used the five items that were a part of the Preoccupation with God dimension (e.g., “*I think about God all the time*”). Participants indicate their answer on a five-point Likert scale, answers ranging from “*strongly disagree*” (1), to “*strongly agree*” (5). The higher a participants' total score is, the more increased is the preoccupation with religiosity. Cronbach's alpha for the scale was 0.94 for the present situation and 0.95 for the past situation in the present study.

### Procedure

A cross-sectional web-based survey design was adopted. The study was completed online during Romania's national lockdown in the period April 21–30, 2020, anonymously and voluntarily, in the participants' native language of instruction. Participants completed UCLA Loneliness Scale, Preoccupation with God, and DOS twice, once thinking about the present situation during the coronavirus pandemic, and once referring to the way they would have answered before the pandemic. They completed Fear of COVID-19 once and, finally, provided demographic information. The Ethics Committee of the Faculty approved the study.

### Data Analysis

Statistical analyses were performed using the SPSS (Version 25) and MPLUS (Muthén and Muthén, 2016) in case of the factor analyses. No missing data were detected. Prior to data analysis, data were tested for normality.

Confirmatory factor analyses were performed using the robust maximum likelihood (MLR) estimator. Model fit was assessed based on Comparative Fit Index (CFI), Root-Mean-Square Error of Approximation (RMSEA), and Standardized Root-Mean-Square Residual (SRMR). Cutoff scores were 0.08 for RMSEA and SRMR and 0.90 for CFI and TLI (Browne and Cudeck, 1993; Hu and Bentler, 1998; Schreiber et al., 2006).

Configural, measurement, and scalar invariance was assessed across different age groups (young adult: 18–34, middle adult: 35–50, older adult: 50+), gender (male, female), knowing someone with COVID-19 (yes, no), and preexisting condition (no dangerous preexisting condition, chronic illness dangerous for COVID-19). Models were compared with the Satorra-Bentler scaled χ^2^ difference tests using scaling correction factors, together with CFI, RMSEA, and SRMR differences. As χ^2^ is sensitive to sample size, final decisions were made based on the fit indices. In our adequately large sample, changes of ≤-0.010 for CFI and a change of ≤0.015 for RMSEA and a change of ≤0.010 for SRMR indicated that the invariance holds (Chen, 2007).

Descriptive statistics (means, medians, standard variations) Cronbach's alphas and Spearman's correlation coefficients were calculated for continuous variables. Dichotomously categorized variables were compared with continuous variables by employing Pearson's point biserial correlation coefficient. Relationships between categorical variables were assessed with χ^2^-tests.

Linear regression analysis was performed to test the effects of the study variables on Fear of COVID-19. For these analyses, data were centralized. Due to multicollinearity, past scores of loneliness, death obsession, and religiosity were not included in the model, all variance inflation factors then remained below two. Demographic variables, mean and difference scores of the formal questionnaires together with their interactions with age, gender, and other questionnaire scores were included in the analysis. Effect sizes *r* of 0.10, 0.30, and 0.50 were considered small, medium, and large, respectively (see Cohen, 1988). Significant interactions were followed by simple slope analyses via PROCESS (version 3.5) without covariants. In these analyses, simple regression slopes were tested between the predictor and the outcome variable at low (1 SD), mean (0 SD), and high (−1 SD), levels of the moderating variable.

## Results

### Confirmatory Factor Analysis and Internal Consistency of the Fear of COVID-19 Scale

The initial factor solution of all seven items loading on one factor yielded to unsatisfactory results, χ^2^ = 306.30, *df* = 14, *p* < 0.001, CFI = 0.896, TLI = 0.844, RMSEA = 0.125 [90% CI 0.113, 0.137], SRMR = 0.052. After assessing the modification indices, error terms for items 1 and 2 and for items 6 and 7 were allowed to correlate. This solution was deemed to be acceptable, χ^2^ = 114.65, *df* = 12, *p* < 0.001, CFI = 0.964, TLI = 0.936, RMSEA = 0.080 [90% CI 0.067, 0.094], SRMR = 0.035. Configural, metric, and scalar invariance was tested among age groups, gender, knowing someone with COVID-19 and preexisting condition. In all multi-group analyses, the invariance held (see Table 1).

**Table 1:** Goodness-of-fit statistics for the multi-group invariance testing of the Romanian Fear of COVID-19 scale.

**Model**	**Comparison**	**χ^**2**^**	***p***	***df***	**Δχ^**2**^ (scaled)**	**Δ*df***	***p* for Δχ2**	**RMSEA**	**ΔRMSEA**	**CFI**	**ΔCFI**	**SRMR**	**ΔSRMR**
**Age: 18–34 (*****n*** **=** **473), 35-45, (*****n*** **=** **435), 50+(*****n*** **=** **432)**													
1. Configural	–	149.72	<0.001	36	–	–	–	0.084 [CI 0.070, 0.098]	–	0.961	–	0.037	–
2. Metric	1	168.91	<0.001	48	16.29	12	0.178	0.075 [CI 0.063, 0.088]	0.009	0.958	0.003	0.047	0.010
3. Scalar	2	205.70	<0.001	60	35.68	12	<0.001	0.074 [CI 0.063, 0.085]	0.001	0.950	0.008	0.047	0.000
**Gender: female (*****n*** **=** **1125), male (*****n*** **=** **215)**													
4. Configural	–	129.30	<0.001	24	–	–	–	0.081 [CI 0.068, 0.095]	–	0.963	–	0.035	–
5. Metric	4	135.84	<0.001	30	7.56	6	0.272	0.073 [CI 0.060, 0.085]	0.008	0.963	0.000	0.038	0.003
6. Scalar	5	16.26	<0.001	36	19.33	6	0.004	0.072 [CI 0.061, 0.083]	0.001	0.957	0.006	0.043	0.005
**Know someone with COVID-19: yes (*****n*** **=** **944), no (*****n*** **=** **396)**													
7. Configural	–	13.64	<0.001	24	–	–	–	0.081 [CI 0.068, 0.095]	–	0.963	–	0.036	–
8. Metric	7	14.21	<0.001	30	11.57	6	0.072	0.074 [CI 0.062, 0.087]	0.005	0.962	0.001	0.038	0.002
9. Scalar	9	153.64	<0.001	36	1.40	6	0.109	0.070 [CI 0.059, 0.081]	0.004	0.959	0.003	0.042	0.004
**Preexisting condition dangerous for COVID-19: no (*****n*** **=1196), yes (*****n*** **=144)**													
10. Configural		134.01	<0.001	24	–	–	–	0.083 [CI 0.069, 0.097]	–	0.964	–	0.036	–
11. Metric	10	149.74	<0.001	30	10.71	6	0.098	0.077 [CI 0.065, 0.090]	0.006	0.961	0.006	0.042	0.006
12. Scalar	11	169.90	<0.001	36	19.10	6	0.004	0.075 [CI 0.063, 0.086]	0.002	0.956	0.005	0.041	0.001

Internal consistency of the measure was adequate with a Cronbach's alpha of 0.87. Item-total correlations were 0.78, 0.78, 0.65, 0.81, 0.76, 0.70, 0.78, for items 1 to 7, respectively.

### Descriptive Analysis of the Study Variables

As it can be observed in Table 2, most of the participants were female and not a student. The majority did not have any preexisting condition dangerous to COVID-19 nor did they know someone with COVID-19. The average isolation time was 37.07 days (*SD* = 165.34) and most of the participants maintained their usual daily activities during this time (74.6%, see Table 2).

**Table 2:** Descriptive statistics and relationships between variables.

	**1**	**2**	**3**	**4**	**5**	**6**	**7**	**8**	**9**	**10**	**11**	**12**	**13**	**14**	**15**	**16**	**17**
1. Fear of COVID-19																	
2. Current loneliness	0.220**																
3. Past loneliness	0.200**	0.887**															
4. Loneliness difference	0.026	0.030	0.432**														
5. Current preoccupation with God	0.132**	−0.015	0.011	0.095**													
6. Past preoccupation with God	0.092**	−0.025	0.004	0.093**	0.944**												
7. Preoccupation with God difference	−0.092**	−0.015	0.007	0.041	0.085**	0.358**											
8. Current death obsession	0.533**	0.296**	0.280**	0.055*	0.114**	0.091**	−0.049										
9. Past death obsession	0.408**	0.292**	0.313**	0.126**	0.115**	0.105**	−0.021	0.764**									
10. Death obsession difference	−0.222**	−0.064*	−0.010	0.091**	0.007	0.031	0.057*	−0.392**	0.154**								
11. Age	0.064*	−0.143**	−0.114**	0.047	0.123**	0.117**	−0.031	0.051	0.078**	0.036							
12. Isolation time	0.078**	0.031	0.026	−0.001	0.026	0.015	−0.021	0.005	0.018	−0.025	−0.176**						
13. Gender	−0.127**	0.003	0.004	0.002	−0.149**	−0.126**	0.042	−0.032	−0.042	−0.012	−0.085**	−0.017					
14. Occupation	0.066*	−0.085**	−0.070*	0.018	0.058*	0.033	−0.063*	0.010	−0.004	−0.024	0.335**	0.002	0.184				
15. Preexisting condition	0.127**	0.042	0.049	0.023	0.083**	0.073**	−0.015	0.121**	0.099**	−0.047	0.216**	−0.012	1.505	5.070*			
16. Maintained usual daily activities	−0.130**	−0.137**	−0.139**	−0.031	−0.087**	−0.077**	0.014	−0.037	−0.026	0.021	0.042	0.000	1.669	8.258*	1.716		
17. Know someone with COVID-19	0.099**	−0.011	−0.019	−0.021	0.003	0.002	−0.003	0.047	0.001	−0.077**	0.036	0.034	0.161	6.514*	1.552	1.059	
*M/n*	15.41	40.45	39.22	−1.23	15.66	14.55	−1.10	21.50	20.54	−0.96	38.83	37.07	1125	447	1196	340	944
*SD/*%	5.57	10.10	11.07	5.18	5.97	6.36	2.15	8.58	8.02	5.16	10.86	165.34	84.0	33.4	89.3	25.4	70.4
Cronbach's alpha	0.87	0.92	0.94	N/A	0.94	0.96	N/A	0.94	0.94	N/A	N/A	N/A	N/A	N/A	N/A	N/A	N/A

N = 1,340. Continuous variables were compared with Spearman's rho with each other, dichotomous variables were compared with χ^2^-tests with each other and with Pearson's point biserial correlation with continuous variables. For all χ^2^-tests, df = 1. *p < 0.050; **p < 0.001. All p-values are two-tailed.

*Dichotomous variables: Gender: 1 = female, 2 = male; Occupation: 1 = student, 2 = employed/retired; Preexisting condition: 1 = no preexisting condition dangerous to COVID-19, 2 = Chronic illness dangerous to COVID-19; Maintained usual activities: 1 = no, 2 = yes; Know someone with COVID-19: 1 = yes, 2 = no. Data of n (%) refers to category 1 in each case. N/A = not applicable*.

As for the loneliness, death obsession, and preoccupation with God levels, results are comparable with data from previous studies (see Table 2).

Median loneliness levels significantly increased from 38 to 40 after the pandemics (*Z* = −9.43, *p* < 0.001). Similar changes were observed in case of death obsession (medians changed from 17 to 18 points (*Z* = −7.50, *p* < 0.001), and religiosity (medians increased from 15 to 16 points (*Z* = −18.34, *p* < 0.001). These results indicate that the use of difference scores is adequate.

### Relationships Between Study Variables

Relationships among variables can also be observed in Table 2. Significant, positive relationships were observed among death obsession, preoccupation with God, loneliness, and fear of COVID-19.

### Predictors of Fear of COVID-19

The regression model (see Table 3) explained 34% of the variance of the Fear of COVID-19 scale scores. According to this analysis, males showed less fear of COVID-19 than females. Knowing someone with COVID-19 was positively associated with fear while maintaining usual daily activities was negatively related to it. Age, occupation, preexisting condition, isolation time did not affect fear levels. Nevertheless, the preexisting condition had close to significant results at *p* = 0.059, indicating a tendency among people with chronic illness dangerous to COVID-19 to report more fear toward the disease. Similarly, there was a tendency among students to show less fear than their employed counterparts, *p* = 0.061.

**Table 3:** Regression analysis of the Fear of COVID-19 scale.

**Predictors**	**B**	**SE**	**β**	***p***	***r***
Age	0.004	0.013	0.008	0.752	0.007
Gender	−1.447	0.359	−0.095**	0.000	0.090
Occupation	0.528	0.282	0.045	0.061	0.042
Preexisting condition	0.784	0.415	0.044	0.059	0.042
Isolation time	0.000	0.001	−0.008	0.722	0.008
Maintained usual daily activities	−1.135	0.291	−0.089**	0.000	0.087
Know someone diagnosed with COVID-19	0.868	0.274	0.071**	0.002	0.070
Loneliness	0.042	0.013	0.076*	0.002	0.069
Loneliness difference	−0.008	0.024	−0.008	0.729	0.008
Preoccupation with God	0.051	0.022	0.055*	0.020	0.052
Preoccupation with God difference	−0.218	0.058	−0.084**	0.000	0.083
Death obsession	0.311	0.018	0.479**	0.000	0.378
Death obsession difference	−0.062	0.027	−0.057*	0.021	0.051
Age ^X^ Gender	0.083	0.032	0.062*	0.010	0.058
Age ^X^ Loneliness	0.003	0.001	0.051*	0.037	0.046
Age ^X^ Preoccupation with God	−0.003	0.002	−0.040	0.092	0.037
Age ^X^ Death obsession	0.000	0.001	−0.008	0.745	0.007
Gender ^X^ Loneliness	−0.093	0.038	−0.059*	0.016	0.054
Gender ^X^ Preoccupation with God	−0.096	0.059	−0.039	0.103	0.036
Gender ^X^ Death obsession	0.015	0.042	0.008	0.724	0.008
Loneliness ^X^ Preoccupation with God	−0.002	0.002	−0.023	0.343	0.021
Loneliness ^X^ Death obsession	−0.004	0.002	−0.068*	0.007	0.060
Preoccupation with God ^X^ Death obsession	0.002	0.003	0.016	0.509	0.015

N = 1,340; *p < 0.050, **p < 0.001. All p-values are two-tailed.

As for the formal questionnaires, the current loneliness level was a significant predictor of fear of COVID-19 but the increase of loneliness was not. Current levels of preoccupation with God, death obsession, and their difference scores all explained a significant amount of the variance of the Fear of COVID-19.

Several interactions were detected in the model: age and gender; age and loneliness; gender and loneliness; and death obsession and loneliness. Simple slope analyses showed that among younger adults (−1 SD below the mean, below 24, *B* = −2.87, SE = 0.53, *p* < 0.001) and middle adults (between 24 and 35 years old, *B* = −1.66, SE = 0.42, *p* < 0.001), women showed significantly more fear than men. However, no difference between genders was detected among older adults (+1 SD above the average, above 45 (*B* = −0.44, SE = 0.61, *p* = 0.471).

Loneliness levels acted as a moderator between other variables and fear of COVID-19. Among people with low levels of loneliness, the relationship between age and fear of COVID was not significant, *B* = −0.01, SE = 0.02, *p* = 0.429. However, age and fear of COVID had a significant positive relationship among participants with average, *B* = 0.05, SE = 0.01, *p* = 0.001, and high levels, *B* = 0.08, SE = 0.02, *p* < 0.001, of loneliness, showing the strongest relationship among people with high levels of loneliness. These data show that loneliness among older people is a risk factor for showing high levels of fear of COVID-19.

At low loneliness levels, no differences were observed between men and women, *B* = −0.50, SE = 0.58, *p* = 0.382. However, at mean, *B* = −1.93, SE = 0.40, *p* < 0.001, and high, *B* = −3.35, SE = 0.58, *p* < 0.001, loneliness, women reported more fear than men, the difference being the most pronounced at high loneliness levels.

At all loneliness levels, death obsession was a significant predictor of fear of COVID, however this effect was more pronounced at low levels of loneliness, *B* = 0.41, SE = 0.03, *p* < 0.001, than at medium, *B* = 0.35, SE = 0.02, *p* < 0.001, and high levels B = 0.29, SE = 0.02, *p* < 0.001.

## Discussion

This study was designed to gain insight into the psychological effects of the quarantine from an existential perspective in the context of the COVID-19 pandemic and to examine the relationships of loneliness, preoccupation with God, and death obsession with fear of COVID-19. Results showed that after an average isolation time of almost 37 days, the perceived levels of loneliness, death obsession, and preoccupation with God significantly increased in our Romanian sample. Despite the benefits of the quarantine to public health, it also caused psychological damage (Brooks et al., 2020). Other studies showed that people in a state of confinement may express fixation on the disease as well as feelings of loneliness, anxiety, and depression (Brooks et al., 2020; Li et al., 2020). In addition, it also amplified the fear of infecting other people and manifesting symptoms of the disease (Reynolds et al., 2008). Together with our results, these findings indicate that the difficult conditions of the current pandemic generally bring existential issues to the surface such as mortality awareness, the feeling of loneliness, and spiritual/religious concerns, as previously suggested (Wong, 2020a,b). Nonetheless, this study shows that it would be necessary to differentiate those who struggle with these issues to a significant extent from those who do not report a significant impact.

We also found that loneliness, death obsession, and preoccupation with God were positive predictors of fear of COVID-19, supporting the second hypothesis of this study. As more people live alone (Kearns et al., 2015), loneliness and social isolation are generally increasing in society (Holt-Lunstad et al., 2015). However, during quarantine, loneliness may be considered a temporary psychological response to a change in an individual's social environment. Previous research showed that loneliness may act as a stressor (Algren et al., 2020) that produces negative affects, such as higher levels of perceived stress (Kearns et al., 2015). Similarly, mandatory prohibitions against praying in a church during the quarantine compromised the religious lifestyle and increased the anxiety of religious individuals (aChord Center, 2020). In the current study, preoccupation with God was associated with increased fear of COVID-19, while previous studies found that the perception of the coronavirus as a health threat was linked to the phenomenon of religious immunity (aChord Center, 2020). Data from several waves of the World Values Survey showed that 97% of Romanian adults declared that they believe in God and 93% characterized themselves as religious persons (Pickel, 2009). As a consequence, our results suggest that this pandemic-specific situation determined people to be more preoccupied with praying and thinking about God in order to mitigate their level of fear.

Death obsession was the strongest predictor of fear of COVID-19. Interestingly, obsessive thoughts have been conceptualized as being functionally associated with symptoms of fear (Salkovskis, 1985). In the context of the pandemic, individuals with intrusive thoughts centered on their own death and that of significant others have an increased fear of COVID-19. This result is consistent with previous studies that explored the relationship between death obsession and coronavirus perceived risk (Yildirim and Güler, 2021). This study indicates that death anxiety is clearly the existential angst most related to the COVID-19 spread. Therefore, in line with Wong (2020b), these findings highlight the significance of promoting death acceptance (Wong, 2008). As such, it is more important than other strategies like treating the feelings of loneliness and religious beliefs to effectively cope with the anxiety and intense fear produced by the COVID-19 pandemic. Moreover, the relationship between death obsession and fear of COVID-19 was moderated by the level of loneliness. Death obsession was a greater predictor of fear of COVID-19 among people with lower levels of loneliness compared to individuals with higher levels of loneliness. The reason behind this moderation effect is that as loneliness levels increase, death obsession also increases. However, there is a less relevant relationship between loneliness and fear of COVID-19. Some individuals that communicate with others infrequently do not feel lonely, while others may feel lonely despite having frequent social interactions (Algren et al., 2020).

Our findings also indicate that women are more afraid of coronavirus than men. This result is in line with previous studies that indicate a higher fear of infection in women compared to men (Bitan et al., 2020; Savitsky et al., 2020). The relationship between gender and fear of COVID-19 was exacerbated by loneliness, that is, with higher levels of loneliness, women show even more fear than men. Age was not a direct predictor of fear of COVID-19, although we found a positive low correlation between these variables. Loneliness moderated the relationship between age and fear of COVID-19, meaning that when loneliness levels are high, age becomes a significant predictor of fear of COVID-19: older people are more afraid than younger individuals when they feel lonely.

The existential struggles in this study are approached from existential positive psychology (Wong, 2011, 2020a), a recently developed paradigm that integrates the positive and negative aspects of living as the foundation of well-being. According to Wong (2020a,b), the COVID-19 pandemic has brought to the surface several existential issues such as feelings of loneliness, mortality awareness, and a greater preoccupation with spirituality. Although these struggles are usually linked to increased fear of the situation, they can also act as promoters of personal growth and resilience. Based on Viktor E. Frankl work (1984, 1988), Wong (2020b) proposes a meaning-centered coping style to face this pandemic in a resilient transformative way. This existential coping style is composed of toughness (responding with courage and fortitude to problems and risks), responsibility (being accountable for oneself and others), appreciation (of life and daily experiences), mindfulness (accepting the situation with openness and without judgment), meaning (positive reframing, transforming adversity by seeking self-transcendence), and belief (maintaining faith and hope for a better future).

Concerning the adaptation of the FCV-19S proposed by Ahorsu et al. (2020) into the Romanian language, the results showed that this scale is a reliable and valid measurement tool for assessing fear of COVID-19 among a normative population of participants in Romania. The scores obtained on the FCV-19S were medium-level or moderate, being similar to the results of Martínez-Lorca et al. (2020) and Reznik et al. (2020) in Europe, and in contrast to Ahorsu et al. (2020) and Sakib et al. (2020), who reported high scores. Romanian FCV-19S had satisfactory concurrent validity, as evidenced by the significant association with relevant demographics. The construct validity of the scale confirms a unifactorial structure with good internal consistency reliability. This result is in accordance with the original study (Ahorsu et al., 2020) and in contrast to Bitan et al. (2020), who proposed a two-factor model for the Hebrew FCV-19S. The scale also demonstrated good discriminant and convergent validity as evidenced by the significant correlations with death obsession, loneliness, and preoccupation with God during the quarantine. However, this study did not examine the stability of the scale over time and future studies should incorporate test-retest reliability measures.

The findings of this study should be considered in light of some limitations. Most importantly, effect sizes were small, meaning that although the measured variables were significant predictors of fear of COVID-19, there might be other more prominent factors that future studies ought to assess. Due to the cross-sectional design of our study with self-reported measures, data do not allow us to establish causality. Future research using longitudinal design during a quarantine period is likely to provide important insights into causal relationships among variables investigated in this study. Further attention should also be given considering the frequency of social interaction via the different social technologies (Algren et al., 2020) and the number of family members, friends, and others when we measure loneliness. Our findings highlight the importance of psychological interventions targeting the experience of loneliness and spiritual beliefs in order to promote death acceptance.

## Data Availability Statement

The raw data supporting the conclusions of this article will be made available by the authors, without undue reservation.

## Ethics Statement

All procedures performed in this study involving human participants were in accordance with the ethical standards of the institutional research committee of Alexandru Ioan Cuza University and with the 1964 Helsinki declaration and its later amendments or comparable ethical standards. The participants provided their written informed consent to participate in this study.

## Author Contributions

VE developed the initial research question and designed the study. NE contributed to the data analysis. TP collected the data. VE, NE, TP, and DC wrote the manuscript, improved, and approved the final version of the paper. All authors contributed to the article and approved the submitted version.

## Conflict of Interest

The authors declare that the research was conducted in the absence of any commercial or financial relationships that could be construed as a potential conflict of interest.
